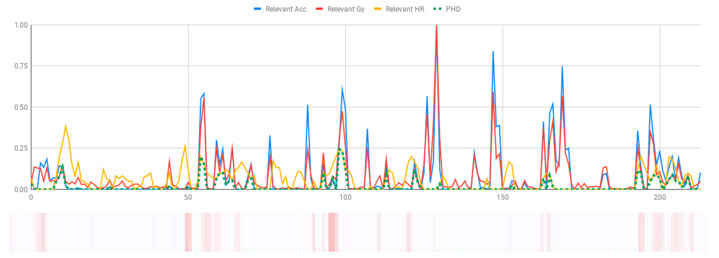# Fuzzy Protoform for Hyperactive Behaviour Detection Based on Commercial Devices

**DOI:** 10.3390/ijerph17186752

**Published:** 2020-09-16

**Authors:** Antonio-Pedro Albín-Rodríguez, Adrián-Jesús Ricoy-Cano, Yolanda-María de-la-Fuente-Robles, Macarena Espinilla-Estévez

**Affiliations:** 1 Education and Sports Council, Junta de Andalucía (Regional Government of Andalusia), 23007 Jaén, Spain; antoniop.albin.edu@juntadeandalucia.es; 2 Social Work Department, University of Jaén, 23071 Jaén, Spain; adrian.ricoy@gmail.com (A.-J.R.-C.); ymfuente@ujaen.es (Y.-M.d.-l.-F.-R.); 3 Computer Science Department, University of Jaén, 23071 Jaén, Spain

**Keywords:** hyperactive behaviour, inertial measurement units, optical heart rate sensor, wearable devices, commercial device, fuzzy protoform, temporal windows, aggregation operator, fuzzy linguistic terms

## Abstract

Hyperactive behaviour refers to a person making more movement than expected for his or her age and development, acting impulsively, and being easily distracted. There is a need to encourage early and reliable detection through the proposal of new methodologies and systems in the context of hyperactive behaviour to prevent or lessen related problems and disorders. This paper presents a methodology to compute a fuzzy protoform (a linguistic description) as an estimator for hyperactive behaviour. The proposed methodology is developed in a system called Smart HyBeDe, which integrate non-invasive and commercial wearable devices, such as activity bracelets, in order to capture data streams from inertial measurement units and optical heart rate sensors. The generated data by the wearable device are synchronized with a mobile device to process the fuzzy protoform to inform family members and professionals. Three datasets generated by the wearable device in real contexts are presented. These datasets are used to evaluate the impact of wrist choice for the wearable device, multiple fuzzy temporal windows, different aggregation operators, and relevant linguistic terms to define the fuzzy protoform as an estimator for the hyperactive behaviour. The results, analysed by a hyperactive behaviour expert, show that the proposed protoform is a suitable hyperactive behaviour estimator.

## 1. Introduction

Hyperactive behaviour is a neurobiological disorder in which a person moves more than expected for his or her age and maturity, acts impulsively, and is easily distracted [1]. Since this behaviour occurs more frequently in school-aged adolescents, its detection is key. If they are not diagnosed and treated early, they are at great risk for major dysfunctions in adulthood [2], such as developing an attention-deficit hyperactivity disorder, brain or central nervous system disorders, emotional disturbances, or hyperthyroidism [3].

Early detection of hyperactive behaviour is vitally important, especially in school-aged adolescents, as it has a negative influence on different areas if not carried out in time. The most prominent problems, among others, are poor educational achievement, negative impact on affective-social relationships [4], learning difficulties, and, lastly, problems resulting from inappropriate behaviour [5].

To assess hyperactive behaviour in school-aged adolescents, the following tools have been used: the Diagnostic and Statistical Manual of Mental Disorders (DSM-5) [6] and the Conners rating scales [7] in which the latter is the most widely used by professionals.

On the one hand, the Diagnostic and Statistical Manual of Mental Disorders (DSM-5) serves as a reference for health professionals in the diagnosis of this type of disorder. Section F90 of the DSM-5 manual, on attention deficit disorders, includes a set of symptoms of hyperactive behaviour, such as: (i) often fidgets with or taps hands or feet or squirms in seat, (ii) often leaves the seat in situations when remaining seated is expected (e.g., leaves his or her place in the classroom, in the office or other workplace, or in other situations that require remaining in place), (iii) often runs about or climbs in situations where it is inappropriate (note: in adolescents or adults, this may be limited to feeling restless), or (iv) often unable to play or engage in leisure activities quietly. According to the DSM-5, six (or more) of the symptoms must be present for hyperactive behaviour to be considered in adolescents under the age of 17. For older adolescents and adults (17 years of age and older), a minimum of five symptoms are required [8].

On the other hand, the Conners rating scales assess the hyperactive behaviour of adolescents under 17 years of age through information collected by their parents and teachers in a given questionnaire [7]. As an example, the questions for teachers on the original scale (CTRS-39) related to the hyperactivity factor include descriptions such as: (i) excitable, impulsive, (ii) restless, (iii) disturbs other children, or (iv) temper outbursts and unpredictable behaviour. The questions in Conners rating scales are scored on a numerical scale where 0 is *Never*, 1 is *Rarely*, 2 is *Sometimes*, and 3 is *Very often.* Once the scores given to the questions have been added up, a final score is obtained, which will correspond to an index of hyperactivity, according to the age and gender of the adolescent.

This kind of diagnosis is strongly marked by imprecision, vagueness, and the subjectivity of the tests [9], as based on perceptions of the adolescent’s environment. This fact has been proven to lead to erroneous diagnoses, which has important consequences [10].

There is a new trend of more reliable diagnostic methods based on the collection signals by wearable devices that integrate Inertial Measurement Units (IMUs) or Optical Heart Rate (OHR) sensors. These devices generate data of acceleration, rotation, and heart rate to obtain key indicators in multiple domains such as Parkinson’s disease [11,12,13], fitness coaching [14], insomnia stage [15], fall detection [16], lumbosacral gait [17], or activity recognition [18].

The proposed systems in the literature that integrate IMUs and OHR sensors [19,20] in the context of hyperactive behaviour have the following shortcomings [21,22,23,24]: (i) the need to carry a large number of IMUs distributed throughout the body, resulting in invasiveness [25], (ii) they limit a person’s movement and activity, so people cannot perform their functions and tasks in a natural way [26], (iii) it is not easy to deploy the system on people’s bodies and they are overly dependent on external power sources [27], (iv) there is a lack of systems to provide relevant follow-up reports over long evaluation periods [23], (v) it is tested in simulated environments and not in real environments where the person acts naturally [27], and (vi) commercial options are expensive and, despite this, do not cover all needs [28].

This paper starts with the hypothesis that the proposal of a system for monitoring hyperactive behaviour with wearable and mobile devices, would offer a more reliable and accurate means of computing a fuzzy protoform as a hyperactive behaviour estimator. This behaviour estimator would be obtained by processing the data streams of IMUs and an OHR sensor of the wearable device in order as to acquire more accurate monitoring in the person’s everyday environment.

From the field of soft computing [29], Fuzzy protoforms [30,31] were proposed as a successful tool for modelling inaccurate data. Therefore, they have been proposed in multiple applications such as activity recognition, health of the elderly, early illness detection, or cardiac rehabilitation to model sensor data for their inaccuracy due to calibrations, lack of battery, and transmission errors in the network [32,33,34,35,36].

Fuzzy protoforms are linguistic descriptions of data [37] that convey the most relevant information contained and are, sometimes, hidden in them. Fuzzy protoforms were proposed by Zadeh [38,39] as a useful knowledge model for reasoning [40], summarization [41], and aggregation [42] of data under uncertainty. Data are modeled by fuzzy sets whose degree of truth to fuzzy sets is defined by membership functions.

To do so, this paper proposes a methodology based on fuzzy temporal windows, aggregation operators, and fuzzy relevant linguistic terms to define a fuzzy protoform as an estimator for the hyperactive behaviour, which fuse acceleration, gyroscope, and heart rate data. Fuzzy protoforms have been proven successfully in other contexts with sensors, such as in cardiac rehabilitation [43,44], in-home sensor data [45], in daily pulse rate measurements of an elderly resident [46], in energy consumption time series set [47], in eldercare [48], and in activity recognition [18,49,50,51,52].

The proposed methodology is deployed in a system called Smart HyBeDe (Hyperactive Behaviour Detection) using commercial devices. Additionally, three datasets are presented in three different real environments where four people with different behaviour are involved. Lastly, the evaluation of the methodology is carried out by an expert in hyperactive behaviour to assess the wrist to wear the device, fuzzy temporal windows, aggregation operators, and relevant linguistic terms to present the proposed fuzzy protoform as an estimator for the hyperactive behaviour.

The remainder of this paper is structured as follows. Section 2 presents a review of works related to the latest trends in technologies for the detection of hyperactive behaviour by emphasizing the main novelties of the proposal presented in this paper. Section 3 presents the proposed fuzzy protoform to obtain a hyperactive behaviour estimator. Section 4 presents the Smart HyBeDe (Hyperactive Behaviour Detection) system, which implements the proposed methodology as well as three datasets generated with the system. Section 5 presents the evaluation of three cases to obtain, with expert input, a fuzzy protoform to obtain the hyperactive behaviour estimator. Lastly, in Section 6, conclusions and ongoing works are discussed.

## 2. Related Works

In this section, the most relevant systems presented in the literature are reviewed. These systems have been designed with wearable devices that help provide a more accurate diagnosis of hyperactive behaviour estimators than traditional tools based on subjective interpretation [53,54].

### 2.1. AULA NESPLORA System

A virtual reality system was proposed in Reference [27] that simulates scenarios in order to stimulate different sensory channels through virtual reality glasses, AULA NESPLORA, used in the evaluation of sustained attention, hearing, and vision as well as impulsivity and hyperactivity in children between 6 and 16 years of age [28]. The system records the responses to the stimuli, shown through the glasses, by means of a button that the patient activates when he or she recognizes the stimulus, and from the data collected on acceleration and spinning located on the glasses by means of IMUs [55].

The rates taken into account by the system are selected according to the patient responses to the stimulus. These main indexes are: total omissions, total commissions, average response time on hits, standard deviation of response time on hits, and motor activity. Regarding these rates, and in relation to our research, a low response time is linked to higher hyperactivity, and, as for the motor activity, it measures head movements while the patient performs the simulation through the gyroscope of the virtual reality glasses. As a result of the simulation, a statistical analysis of the responses given by the child by button activation is carried out, which establishes reference values to determine whether the performance for each of the indexes has been very low, low, normal, high, or very high.

Despite its usefulness, the system has major drawbacks. First, the system is in the hands of the professional and the patient must go to an appointment with the doctor or physician to measure their behaviour during a limited period of time. In addition, the scenario presented to the patient is simulated and is not a real scenario where the patient’s behaviour can be more accurately measured. Consequently, from the above, it is only possible to capture the patient’s behaviour in the simulated environment by obtaining reports exclusively from that period. Therefore, the reports may not be entirely conclusive as they are focused on a period of time and in a fully controlled and simulated environment. Lastly, it should be noted that the system partially and automatically measures the individual’s response since it is the individual who has to activate the button with which the statistics for the report are obtained.

### 2.2. Broad Set of Wearable Devices with Multiple Sensors

In Reference [24], a system composed of a broad set of wearable devices with sensors located in multiple parts of the subject’s body was proposed. It included IMUs to obtain acceleration data, an OHR sensor [19], and galvanic skin response sensors [56] that support the diagnosis of hyperactive behaviour. The system was used in a closed and controlled environment simulating 11 diagnostic scenarios, according to the DSM-5 manual, and collected the data obtained by the sensors when the subject is interacting with each scenario. It should be noted that this system only collects data without any processing. It does not propose rates of hyperactivity and, therefore, does not offer reports that help professionals.

This system has been proposed to the scientific community and, so far, the information collected by the sensors has not been processed or validated by a clinical professional. Similarly, the system analysed presents significant problems of invasiveness. Another important fact that stands out is that this type of wearable device must be attached to specific areas in order to generate data correctly and reliably during an interaction with the defined scenarios. This fact limits the movements and activities of the person.

### 2.3. Wearable Device with A IMU by Kam

A system similar to the one presented by Reference [24] but simpler is presented in Reference [23], which consists of a single IMU that collects acceleration data on the person, according to their movements. Subsequently, an analysis of this data was carried out and, using artificial intelligence techniques based on decision trees, the person’s activity was classified into two levels: high and low. Lastly, this classification was contrasted with the clinical evaluations carried out by a health professional by obtaining results of more than 98% accuracy.

This system has not been launched on the market, but it has been evaluated in real school settings under the supervision of health professionals by concluding that it can be valid for evaluating hyperactive behaviour. However, it lacks an integrated clinical decision support system, which would allow monitoring of the person’s hyperactive behaviour by the clinician and generation of relevant reports.

### 2.4. Wearable Device with an IMU by Amado

A methodology for the diagnosis of hyperactivity behaviour was proposed in Reference [9] by using wearable and non-intrusive devices with an IMU. Initially, the system trains a model with deep learning techniques by using previously obtained and classified acceleration data, which indicates the absence or presence of hyperactivity. The model allows collection of acceleration input from the subject, outside the device, and by classifying their hyperactivity.

Although this proposal presents a relevant use of artificial intelligence techniques for the processing of data collected by sensors and a subsequent detection of hyperactivity, it is still far from providing a decision support system in the context of hyperactivity.

Firstly, it should be noted that the processing is done offline, which means that the device is on the subject in a real scenario, but neither the information nor the detection is processed in real time. Another important weakness is that a prototype of this device has been designed by the research team, but there is no commercial final product that can be used under the proposed system.

## 3. Methodology

In this section, a new methodology is presented to process and compute a fuzzy protoform to detect a hyperactive behaviour. The proposed methodology integrates a wearable device with IMUs and an OHR sensor to collect the sensor data streams and a mobile device with the aim of overcoming the shortcomings of the reviewed systems based on wearable devices.

To do so, first, we present the system architecture and its components in Section 3.1. Then we present the processing of the raw data from the three data sensor streams in Section 3.2 and, lastly, we present the process to obtain a fuzzy protoform to detect a hyperactive behaviour by means of aggregation operators and temporal windows in Section 3.3.

### 3.1. System Architecture

**Figure 1:**
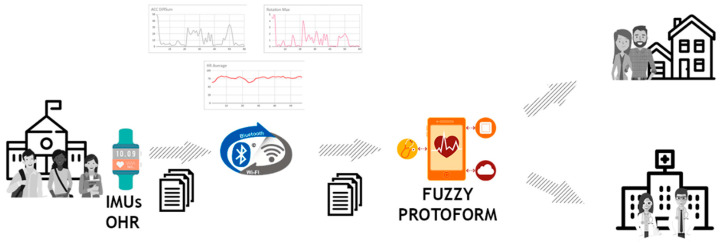
System architecture with a wearable device and a mobile device.

The proposed architecture is based on two applications that are deployed on commercial devices in which the cost allows access to the general public. Specifically, the system is composed of two components: an application installed in the wearable device and an application installed in the mobile device. The description of the architecture is illustrated in Figure 1 and described in detail as follows.

- The wearable device integrates IMUs and an OHR sensor with wireless connectivity capabilities (Bluetooth, Wi-Fi, 4G, etc.). An Android application is installed in this device to collect raw data and record them on the wearable device in separate session files with a common structure. These files are synchronized with the mobile device to compute the proposed fuzzy protoform as an estimator of hyperactive behaviour. Therefore, the main objective of this application is to collect acceleration, rotation, and heart rate data in session files with a common structure.
- The mobile device with wireless connectivity has installed an Android application to synchronize the session files with the common structure stored on the wearable device. The application of the mobile device computes values of a fuzzy protoform that represent the estimator of hyperactive behaviour to provide support to the family of the subject as well as to the professionals. To do so, the Android application integrate a processing based on each data stream by means of low-pass filtering techniques, standardization, and aggregation through temporal windows that will be described in the following subsections. Therefore, the main objective is focused on synchronizing the files generated by the wearable device and perform the proposed computations in order to obtain a value of the hyperactive behaviour estimator.

The main innovations of this architecture, which overcomes the limitations of current systems, are below.

- The person only wears two everyday commercial devices, which integrate with IMUs and an OHR sensor, available at an affordable price. The two devices are a wearable device on the wrist (smartwatch) and his/her mobile device. This paper proposes the use of these devices with Android operating systems due to their popularity and the ability to develop our own applications.
- Android-based wrist devices are capable of operating autonomously for several days, so they are not overly dependent on external power sources.
- The wrist device with IMUs and an OHR sensor collects the data is non-invasive, which allows for natural movement and activity.
- Monitoring of hyperactive behaviour is performed in real environments where the person acts in a natural way.
- The mobile device, through synchronization and the proposed processing, allows us to obtain a fuzzy protoform as an estimator of hyperactive behaviour, which provides monitoring over long periods of time.

Lastly, it is noteworthy that the application of the wearable device of Smart HyBeDe system can also be used as a successful tool to generate datasets of acceleration, rotation, and heart rate data, which the literature has shown its importance to detect hyperactive behaviour.

### 3.2. Model of Hyperactive Behaviour in the Wearable Device

The wearable device obtains data streams from IMUs and an OHR sensor that have been shown to be relevant in measuring hyperactive behaviour (acceleration, gyro, and heart rate data) [9,23,24,53,54].

The collected data is stored sequentially in files, which we will call session files, F_Si_. This will store the samples for a specific time (ST). Furthermore, it is necessary to establish the sampling frequency of the three sensor data streams: acceleration, gyro, and heart rate, which we shall refer to as F_ACC_, F_GY_, and F_HR_. In the session files, each sample collected is defined by S_i_, which will be stored in a row (see Table 1) with the following fields: Type, timestamp, V1, V2, and V3, which are detailed below.

- Type. It indicates the type of measurement generated by the sensor, which can be: ACC (acceleration), GYR (gyroscope), or HR (heart rate).
- Timestamp. It indicates the date and time of the measurement generated by the sensor.
- V1, V2, and V3. These values depend on the type of sensor measured.
  ○ Acceleration: V1, V2, and V3 correspond to the acceleration, respectively, on the X, Y, and Z axis.
  ○ Gyroscope: V1, V2, and V3 correspond to the rotation, respectively, on the X, Y, and Z axis.
  ○ Heart rate: The value V1 corresponds to the heart rate measured by the sensor while the values V2 and V3 are not applicable.

These session files are generated with the raw data streams, which are then synchronized with the mobile device through wireless connectivity in order to process. Only the new session files are synchronized to the mobile device. Both files remain on the mobile device and on the wearable device.

### 3.3. Model of Hyperactive Behaviour in the Mobile Device

In this section, we describe the processing performed on the mobile device with the aim of monitoring hyperactive behaviour. To do this, we first describe the processing performed on the acceleration and rotation data through a low-pass filter in Section 3.3.1. Next, we present the processing to obtain a single standard value of these two signals (acceleration and rotation) in Section 3.3.2. Lastly, we present a fuzzy protoform as an estimator for the hyperactive behaviour by using fuzzy logic, temporal windows, and aggregation operators in Section 3.3.3.

#### 3.3.1. Applying Low-Pass Filter: Acceleration and Rotation

A common feature to all accelerometers and gyroscope sensors that cannot be eliminated, but can be corrected, is the low repeatability of data. It consists of the fact that the data provided by the IMUs fluctuate. Thus, it is necessary to apply a low-pass filtering. Therefore, the current measure is an estimate between the previous values and the one measured at that moment [57,58].

The filtered value at t1 is $\mathrm{fv}1_{t1}$ and is computed by Equation (1). The value generated by the sensor is being represented in timestamp t1 as $v1_{t1}$ and the value of the above is the filtered acceleration represented by $\mathrm{fv}1_{t1-1}$. The parameter alpha is called the smoothing factor and must be between 0 and 1, depending on which measure is to be given the greatest weight in the filtering.

(1) $$\mathrm{fv}1_{t1}=\mathrm{alpha}*v1_{t1}+\left(1-\mathrm{alpha}\right)*\mathrm{fv}1_{t1-1}$$

In Table 2, we show an example of the computed values with a low pass filter with an alpha value of 0.2 for the value v1 of the accelerometer sensor that will be used in this paper [57]. The way in which these fluctuations are reduced is illustrated below.

#### 3.3.2. Applying the Vector Module: Acceleration and Rotation

In this phase, we propose to merge the three acceleration components and the three rotation components through an orientation-independent metric, which is called Magnitude and is defined by Equation (2). Magnitude values are almost stable and are not affected by orientation changes [59].

(2) $$\mathrm{Mag}=\sqrt[{2}]{x^{2}+y^{2}+z^{2}}$$

In addition, to improve the presentation and interpretation of the acceleration data monitoring, the value of gravity is subtracted from magnitude to decrease the range of values.

**Figure 2:**
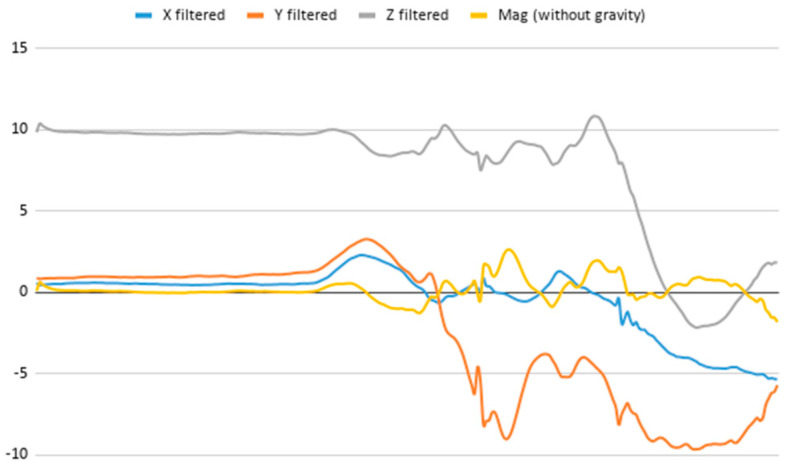
Representation of the filtered X, Y, and Z acceleration values as well as their magnitude without gravity.

An extract of the values processed for acceleration to compute magnitude is represented in Table 3, considering gravity or not. These values are visually represented in Figure 2, where it is interpreted that the person has a calm movement at first, which is followed by rougher movement.

#### 3.3.3. Fuzzy Protoform as Hyperactive Behaviour Estimator

Protoforms are an innovative methodology based on linguistic descriptions to identify relevant or hidden attributes in data streams. In the proposed methodology, we propose a complex protoform as an estimator for the hyperactive behaviour to provide an automated analysis in a comprehensible way. To do so, protoforms are based on fuzzy linguistic terms and fuzzy temporal windows.

A relevant fuzzy linguistic term, µFLT, can be associated with each value of acceleration, gyro, and heart rate by means of a fuzzy trapezoidal membership function µFLT_Acc(acci), µFLT_Gy(gyi), and µFLT_HR(hri), respectively, and can be defined as in Equation (3).

(3) $$µ\mathrm{FLT}\left(X\right)=\mathrm{TF}\left(x\right)\left[l_{1},l_{2},l_{3},l_{4}\right]=\{\begin{array}{cc} 0, & x\leq 0\\ \frac{x-l_{1}}{l_{2}-l_{1}}, & l_{1}\leq x\leq l_{2}\\ 1, & l_{2}\leq x\leq l_{3}\\ \frac{l_{4}-x}{l_{4}-l_{3}}, & l_{3}\leq x\leq l_{4}\\ 0, & l_{4}\leq x \end{array}$$

A Fuzzy Temporal Window (FTW) can be computed to model the sensor data in order to generate weighted fuzzy linguistic terms based on fuzzy temporal linguistic terms and provide flexibility in the presence of uncertainty. An FTW is described in a simple manner according to the distance of the current time t0 to a given timestamp ti as Δti = Δ(|t0 − ti|). In this way, a fuzzy temporal window can be associated with a duration defined in seconds by means of a fuzzy trapezoidal membership function μFTW(Δi) = TF(Δi).

The relevance value vi in a Fuzzy Linguistic Term (FLT) in a fuzzy temporal window is defined by an intersection operation to fuse both degrees of membership by means of Equation (4).

FLT ∩ FTW (vi) = μFLT(vi) ∩ μFTW(Δi) ∈ [0,1](4)

The relevance of a sub-set of the data stream generated by the Wearable Device (WD) associated with an FTW and FLT are aggregated using the union operator in order to obtain a single degree implied in a fuzzy value term in a fuzzy linguistic temporal term by means of Equation (5).

FLT ∪ FTW (WD) = ∪(μFLT(vi) ∩ μFTW(vi)) ∈ [0,1](5)

Therefore, P_0_ represents a basic protoform P_0_(WD) = FLT ∪ FTW (WD) that integrates an interpretable knowledge for the expert in a linguistic way. Basic protoforms can be combined using fuzzy logical operators to increase the linguistic capabilities. In this paper, we propose a protoform composed of basic protoforms that summarize acceleration, gyro, and hear rate data.

## 4. Smart HyBeDe (Hyperactive Behaviour Detection) System

This section presents the Smart HyBeDe (Hyperactive Behaviour Detection) system that implements the proposed methodology and describes three datasets that have been generated with the developed system, and which we used to evaluate our proposals in Section 5.

### 4.1. Description of the Smart HyBeDe System

The proposed methodology has been deployed in a system named Smart HyBeDe (Hyperactive Behaviour Detection), which is based on commercial devices. Specifically, the system is composed of two devices: a wearable device and a mobile device that were illustrated in Figure 1.

Regarding the wearable device, there are currently inexpensive wrist-worn devices available on the market. They allow the collection of data from IMUs and an OHR sensor, depending on the device. In Reference [60], multiple wearable devices were evaluated by highlighting the Polar M600 device, which is why this device was chosen in this paper.

Polar M600 is a highly accurate Android Wear device with a high-quality OHR sensor. The specifications of the Polar M600 are: (1) OHR sensor with 6 LEDs, (2) waterproof (IPX8 10 m), (3) lightweight (63 g), (4) small size (45 × 36 × 13 mm), (5) long-life battery (500 mAh Li-pol for a 2-day average uptime per charge or 8 h of training), and (6) 4GB internal storage [61].

**Figure 3:**
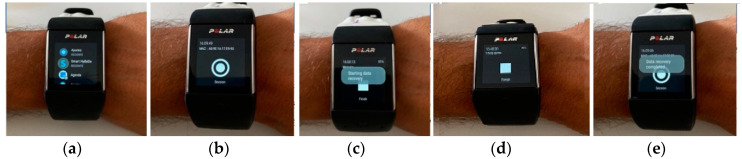
Proposed wearable device, Polar M600, showing: (**a**) the Android Wear application, (**b**) start button to collect the acceleration, gyroscope, and heart rate data, (**c**) start of data collection in the files, (**d**) number of saved files according to the duration of the session, and (**e**) end of session and file saving.

The structure of collected data illustrated in Table 3 was carried out by means of an Android Wear application, as illustrated in Figure 3, installed in the wearable device.

The configuration of the wearable device was set for 5-min session files, which were collected sequentially. The heart rate frequency was 1 Hz and the acceleration and rotation frequency were 50 Hz. These measures have been used with excellent results with this device in the literature [43,44].

Regarding the mobile device, it is the device that processes the data and shows the fuzzy protoform as an estimator for the hyperactive behaviour. In this proposal, BQ Aquaris M5 is chosen as an Android device for its connectivity and processing capabilities as well as its low price [62]. The specifications of the BQ Aquaris M5 are: (1) Android 5.1.1 (Lollipop), upgradable to 7.0 (Nougat), (2) octa-core (4 × 1.5 GHz Cortex-A53 & 4 × 1.0 GHz Cortex-A53) of CPU, (3) lightweight (144 g), (4) small size (143 × 69.4 × 8.4 mm), (5) long-life battery (Li-Po 3120 mAh battery), and (6) internal memory of 16 GB with 2GB RAM [62].

**Figure 4:**
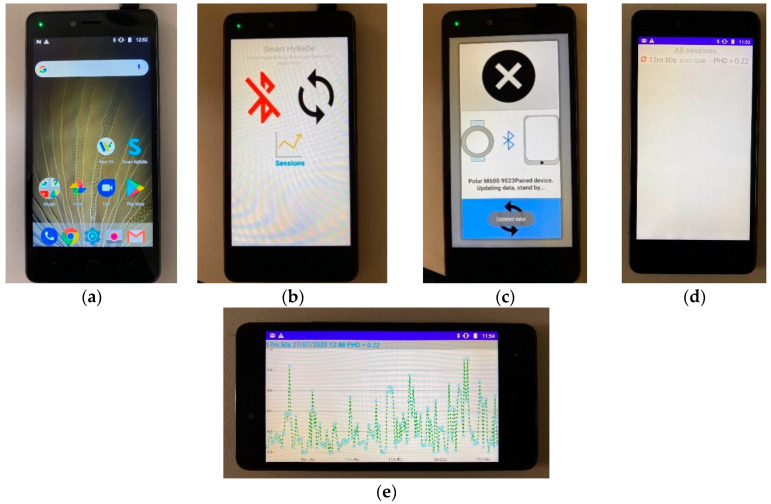
Proposed mobile device, BQ Aquaris M5: (**a**) Smart HyBeDe application installed on the mobile device. (**b**) Main screen showing options of Smart HyBeDe app: pair with a wearable device, synchronize data sessions, and view the processed sessions with the proposed model. (**c**) Synchronization screen getting the new session files from the wearable device. (**d**) Sessions screen in the mobile device and (**e**) display screen of the fuzzy protoform in the mobile device.

A specific Android application was implemented in the mobile device, as part of the Smart HyBeDe system, for data synchronization with the wearable device, and to compute the data to obtain the fuzzy protoform as an estimator for the hyperactive behaviour, as illustrated in Figure 4.

### 4.2. Dataset Generated by the Smart HyBeDe System

The Smart HyBeDe system has been deployed in order to collect data to evaluate the system. To do so, three datasets from four people (A, B, C, D) were collected with the Polar M600 and the Smart HyBeDe system to compute a fuzzy protoform as an estimator for the hyperactive behaviour [63].

Person A participates in all three datasets. This person was evaluated by the expert in hyperactive behaviour as restless and nervous but without highly hyperactive behaviour. This subject is key in the evaluation of estimators since they do not have a distribution of extremes (very calm or very active). This person will be compared in different scenarios.

These datasets are composed of a data set obtained through the IMUs and an OHR sensor of a wearable device, the Polar M600, using the Smart HyBeDe application. The data collection frequency of IMUs was 50 Hz, and 1 Hz for the OHR sensor. Files included in each folder store the data captured by the sensors of one wearable device every 5 min.

- Dataset A—Wrist choice. Person A (17-year-old female subject) working on a personal computer with two wearable devices including one on each wrist. Data collection was performed on 28 July 2020 over a study session of approximately 15 min. Files included in each folder store the data captured by the sensors every 5 min. One of the wearable devices was placed on the wrist of the non-dominant hand and the other on the wrist of the dominant hand. The two sets correspond to the folders described below.
  ○ 1: Person A (17-year-old female subject), dominant wrist.
  ○ 2: Person A (17-year-old female subject), non-dominant wrist.

Person A was evaluated as restless and nervous, without highly hyperactive behaviour.

- Dataset B—Working at home with computer. Two people (A and B) working on a personal computer with a wearable device on the non-dominant hand (left, the two people are right-handed). Data collection took place on 27 July 2020 during a study session of approximately 20 min. A wearable device was assigned to each person: Person A (17-year-old female subject) and Person B (16-year-old male subject). The wearable device was placed on the non-dominant wrist of each user. The two sets correspond to the folders:
  ○ 1: Person B (16-year-old male subject).
  ○ 2: Person A (17-year-old female subject).

Person A was evaluated as restless and nervous without highly hyperactive behaviour and Person B was evaluated as calm by the expert consultant.

- Dataset C—Group activity. This activity was a group discussion to argue different points of view about a specific topic. Three people (A, C, and D) in the group discussion with a wearable device on their non-dominant hand (left, the three are right-handed). Data collection was performed on 2020/07/28 during a group activity of approximately 95 min. A wearable device was assigned to each user: two female subjects aged 17 (Person A) and 15 (Person D), and a male subject aged 16 (Person C). The three datasets correspond to the folders:
  ○ 1: Person A (17-year-old female subject).
  ○ 2: Person C (16-year-old male subject).
  ○ 3: Person D (15-year-old female subject).

Person A is evaluated as restless and nervous without highly hyperactive behaviour. Person C is evaluated as overexcited and nervous and person D is evaluated as very calm by the expert. Person B does not participate in this dataset.

## 5. Evaluation

In this section, the Smart HyBeDe system that implements the methodology presented in this paper is evaluated by the expert in hyperactive behaviour in three cases studies [63] in order to obtain a fuzzy protoform as an estimator for the hyperactive behaviour. The evaluation will be carried out according to the following three evaluation phases.

(1) Evaluation of the impact of wrist choice for the device and the fuzzy temporal window.
(2) Evaluation of aggregation operators in the selected fuzzy temporal window.
(3) Evaluation of relevant fuzzy linguistic terms for the fuzzy protoform.

### 5.1. Evaluation of the Impact of Wrist Choice for the Wearable Device and the Fuzzy Temporal Window

In this section, we evaluate the most suitable wrist to place the device on the person to obtain the fuzzy protoform as an estimator for the hyperactive behaviour. To do so, “Dataset A—Wrist choice” was selected [63]. In this evaluation, we have analysed the following two files: “hybede_1596018209606”, included in folder 1 (Person A: dominant wrist), and “hybede_1596018206291” in folder 2 (Person A: non-dominant wrist). As mentioned, Person A was evaluated as restless and nervous without highly hyperactive behaviour.

The aim of this case study is to obtain relevant information about the wrist on which the wearable device is worn, such as on the dominant or non-dominant hand, as well as the role of the temporal window parameter to compute the baseline data. The computed data were shown to an expert in hyperactive behaviour in order to analyse the results of Person A.

As can be seen below, Table 4, Table 5 and Table 6 graphically show the average values obtained by the acceleration, gyroscope, and heart rate data, respectively, by taking into account fuzzy temporal windows defined by the following fuzzy trapezoidal functions.

- Around0.5seconds = TS_05(x) = TS(0.4,0.5,0.5,0.5)
- Around1second = TS_1(x) = TS(0.8,1,1,1)
- Around3seconds = TS_3(x) = TS(2,3,3,3)
- Around5seconds = TS_5(x) = TS(4,5,5,5)

For data collected by the acceleration and gyroscope data, the graphs in Table 4 and Table 5 show a greater variation in data obtained by the wearable device located on the wrist of the dominant hand when compared with the data obtained from the wrist of the non-dominant hand.

This fact is based on the fact that, when working on a personal computer, it is the dominant hand that is most often used by the person either to move the mouse or the trackpad, write by hand, press the keys on the keyboard, or pick up objects. Such tasks are specific to the activity the person is carrying out and are not considered relevant in determining hyperactive behaviour. For this reason, the data collected by the wearable device located in the person’s non-dominant hand is considered more relevant to obtain a fuzzy protoform as an estimator for the hyperactive behaviour since the discontinuous variation in the data is not associated with the activity itself carried out in the session, which can provide more relevant information through variations in the acceleration and gyroscope data.

In addition, the value of the fuzzy temporal window parameter has a fundamental role in the analysis and computation of the dataset, as shown in the graphs in Table 5. For small fuzzy temporal window values (0.5 s and 1 s), too much variation is shown throughout the session and is caused by movements of the person that are almost imperceptible and that are not considered significant for the purpose of this study. For this reason, the variations in acceleration are better identified for higher fuzzy temporal window values (3 and 5 s), and are more clearly reflected in the graph of the 5-s fuzzy temporal window.

A similar analysis is obtained for the rotation (gyro) data, which suggests it is preferable to place the wearable device on the non-dominant hand of the person since it is considered to provide more relevant information. Regarding the value of the fuzzy temporal window parameter, a value of 5 s shows more representative gyro movements that help us to obtain a fuzzy protoform to detect a hyperactive behaviour.

For the data collected by the heart rate sensors, Table 6 shows no significant differences between wearing the wearable device on the wrist of the dominant or non-dominant hand.

The heart rate variable is unique to the person and the variations reflected in the graphs are due to the nature of the sensors of the wearable device itself and the precise time at which it takes the value throughout the data collection session.

By the nature of the heart rate signal and the heart rate sensor of the wearable device, the value is measured approximately every second. This is due to the fact that the value given to the fuzzy temporal window does not have a great impact on obtaining key relevant information. However, this value is considered in order to analyse and compute the set of data collected during the session.

Given the results, it is clear that having the wearable device on the wrist of the non-dominant hand is adequate for obtaining a fuzzy protoform to detect a hyperactive behaviour. Furthermore, a value of 5 s for the fuzzy temporal window provides relevant information to obtain hyperactive behaviour estimators, which are differentiating.

### 5.2. Evaluation of Aggregation Operators in the Selected Fuzzy Temporal Window

In this section, we evaluate multiple aggregation operators in the selected fuzzy temporal window at *around 5 s*. Two subjects (Person A and Person B) were involved in a similar activity, such as working at home with a computer to do the evaluation. These data were shown to an expert in hyperactive behaviour in order to analyse the results of Person A, which has been evaluated as restless and nervous without highly hyperactive behaviour, and of Person B, which has been evaluated as calm. The aggregation operators studied are the arithmetic average, the sum of the differences of the values, the maximum, the standard deviation, the standard error, and, lastly, the cumulative sum of values [64].

To do so, “Dataset B—Working at home with a computer” was selected [63]. In this case study, we analyse the file “hybede_1595844588722” included in folder 2 (Person A), where the data captured over 5 min of the session were stored.

Table 7 illustrates the graphs for the multiple aggregation operators applied to the acceleration, gyro, and heart rate of the analysed file.

Having been analysed by the expert, the acceleration and rotation results shown in Table 7 have a great similarity when using aggregation operators focused on data variability, such as the sum of the differences of the values, standard deviation, and standard error, even between these aggregation operators and the maximum value in the selected fuzzy temporal window. Furthermore, the results show the arithmetic average of the values and their cumulative sum are similar to each other.

According to the expert, the arithmetic average and the sum of the differences of the values as aggregation operators have been selected as more appropriate in which the differences of the values operator are more relevant to obtain a fuzzy protoform to detect a hyperactive behaviour.

Regarding the type of aggregation operator for the heart rate results, the arithmetic average operator is the most suitable due to the fact that it shows the variations properly, considering the sum of the differences of the values as a second option.

### 5.3. Evaluation of Relevant Fuzzy Linguitic Terms for the Fuzzy Protoform

In this section, we evaluate the fuzzy linguistic term of *relevant* by using the selected fuzzy temporal window *around 5 s* and the aggregation operator of the sum differences of the values for the acceleration and rotation data and the average for the heart rate data.

The definition of this relevant value allows us to represent the fuzzy protoform to detect a hyperactive behaviour P_HD_ as follows:

*P_HD_: Around 5 s Acceleration IS Relevant AND Rotation IS Relevant and Heart rate is Relevant.*

To do so, we analyse and compare three membership functions for each data type with person A, who are involved in a group activity in the same environment, by selecting the “Dataset C—Group activity” [63]. As mentioned, Person A has been evaluated as restless and nervous without highly hyperactive behaviour.

In this evaluation, we analysed a file of the data set collected by the sensors of the wearable device of the person with a total duration of 5 min. The selected file is Folder 1 (Person A): “hybede_1595931327330”.

Taking into account the three fuzzy membership functions for each type of data illustrated in Table 8, the computed data, which are illustrated in Table 9, were shown to an expert on hyperactive behaviour in order to analyse them.

According to the expert, the adequate membership function that is relevant in each data type are shown in bold in Table 8. Therefore, the degrees of membership of these relevant terms provide an intuitive evaluation for each type of data in wrist-worn wearable devices.

**Figure 5:**
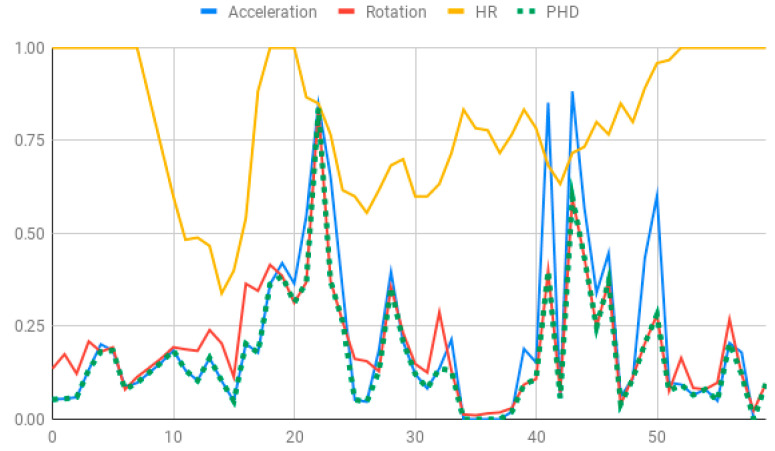
Membership degree to each data type to their relevant value and the membership degree to the protoform as an estimator for the hyperactive behaviour.

The membership degrees of the protoform P_HD_: *Around 5 s Acceleration IS Relevant AND Rotation IS Relevant and the Heart rate is Relevant* are computed by means of the fuzzy union operator, which is the semantic function proposed for the fuzzy union operator as the minimum. Therefore, the membership degrees of the protoform PHD are shown in Figure 5.

Lastly, we analyse four files of the data set collected by each person with the *P_HD_* for 20 min. The selected files are:

- Folder 1 (Person A): “hybede_1595931327330”, “hybede_1595931630361”, “hybede_1595931933797” and “hybede_1595932237796”.
- Folder 2 (Person C): “hybede_1595931337468”, “hybede_1595931641560”, “hybede_1595931946472” and “hybede_1595932251652”.
- Folder 3 (Person D): “hybede_1595931353450”, “hybede_1595931656968”, “hybede_1595931960622” and “hybede_1595932263485”.

According to the evaluated results for the analysis of the data set, the aggregation operator of the arithmetic average and the sum of the differences of the values are used as well as a 5-s temporal window.

Table 10 shows the membership degree of the PHD of the three people doing the same group activity for 20 min. According to the membership degree of the PHD, we gradually change the intensity to evaluate the PHD and to evaluate these sessions.

As can be observed, there are greater membership degrees in the relevance of acceleration, rotation, and heart rate for Person A. This person was evaluated by the expert as restless and nervous without highly hyperactive behaviour. Therefore, the proposed fuzzy protoform to detect a hyperactive behaviour is validated by the expert in which the average membership degree is 0.22.

High peaks of relevant acceleration and relevant rotation are generated for person C, and his heart rate is generally not relevant. This person was evaluated by the expert as overexcited and nervous without hyperactive behaviour. The proposed protoform presents peaks where the estimator is a little higher but not in a regular way throughout the session as in the case of Person A. Therefore, the proposed fuzzy protoform to detect a hyperactive behaviour is validated by the expert in which the average membership degree is 0.12.

The relevance in acceleration, gyro, and heart rate of Person D is extremely low. This person was evaluated as very calm by the expert. Therefore, the proposed fuzzy protoform to detect a hyperactive behaviour is validated by the expert in which the average membership degree of this person is 0.02.

Lastly, it is noteworthy that the computations to obtain the degree of membership of the hyperactive behaviour estimator have been implemented in the mobile device of the Smart HyBeDe system. Thus, Figure 4e illustrates the graph of the estimator values of Person A in the group activity on the mobile device shown in Table 10.

## 6. Conclusions

This paper has presented a methodology based on soft computing to obtain a fuzzy protoform as an estimator for the hyperactive behaviour. This methodology has been developed in a system called Smart HyBeDe, which integrated non-invasive and commercial wearable devices in order to capture data streams from IMUs and an OHR sensor.

The main innovations of the system are that it has been based on commercial devices available at an affordable price. These devices are not overly dependent on external power sources. The data streams are collected with a non-invasive device, which allows the movement and activity of the person in a natural way and, lastly, the monitoring of the person is performed in a real context. Furthermore, Smart HyBeDe provides a tool for the scientific community to generate new datasets in the context of hyperactive behaviour.

The proposed fuzzy protoform has been defined as a hyperactive behaviour estimator by computing the data streams from acceleration, rotation, and heart rate generated by the wearable device. In the presented methodology, a low-pass filter and vector module computations applied to the acceleration and rotation data streams have been included. Furthermore, to fix the fuzzy protoform, fuzzy temporal windows, aggregation operators, and fuzzy linguistic terms were integrated.

Three datasets on four people have been presented, which have been collected by using the Smart HyBeDe system in three different real scenarios and which have been assessed by an expert in hyperactive behaviour. As a result of these evaluations, the following most relevant findings to obtain a fuzzy protoform as a hyperactive behaviour estimator have emerged: (i) the wearable device located on the person’s non-dominant hand offers more key information to obtain the estimator, (ii) the fuzzy temporal window *around 5 s* is adequate to represent the variations in acceleration and rotation. However, the size of the temporal window for heart rate data is not very important since this signal is more stable, (iii) regarding the type of aggregation operator for the acceleration and rotation data, the sum of the differences of the values has been the most suitable due to the fact that it shows the variations properly. For the heart rate, the average has been considered as a first aggregation operator, (iv) the fuzzy linguistic term *relevant* has been defined for each data type, and (v) the fuzzy protoform: *Around 5 s Acceleration IS Relevant AND Rotation IS Relevant and Heart rate is Relevant* has been defined as an estimator for the hyperactive behaviour.

Taking into account the proposal that has been presented in this paper, in future works, we will analyse other interesting estimators for the expert in hyperactive behaviour by considering multiple kinds of activities and their evaluation with attention-deficit hyperactivity disorder (ADHD) subjects with different levels of hyperactive behaviour by means of the proposed Smart HyBeDe system.

## Figures and Tables

**Table 1:** Excerpt from a session file.

Type	Timestamp	V1	V2	V3
GYR	1262368390749	4.503298	−0.791865	−2.995744
GYR	1262368390749	4.51857	−0.835236	−3.018957
GYR	1262368390749	4.498412	−0.939694	−3.018346
HR	1262368390749	64	0	0
ACC	1262368390750	−0.55992	−1.622334	10.41835
ACC	1262368390750	−0.468993	−1.366302	10.9017

**Table 2:** Filtered values computed in acceleration values.

Type	Raw V1	Filtered V1
ACC	0.605384	0.605384
ACC	−0.124427	0.4594218
ACC	0.579063	0.48335004
ACC	0.569492	0.500578432

**Table 3:** Magnitude of the acceleration of the three axis and magnitude without considering gravity.

Type	Filtered V1	Filtered V2	Filtered V3	Mag. Acc.	Mag Acc. -g
ACC	0.605384	0.887737	9.856036	9.914434548	0.1044345478
ACC	0.4594218	0.850409	10.392507	10.43735913	0.6273591297
ACC	0.48335004	0.8636174	10.2665488	10.31414013	0.5041401259
ACC	0.500578432	0.87896972	10.15621104	10.20645821	0.3964582116

**Table 4:** Acceleration results with different fuzzy temporal windows from both wrists.

Acceleration	
Dominant Wrist	Non-Dominant Wrist
Fuzzy Temporal Window = around 0.5 s	Fuzzy Temporal Window = around 0.5 s
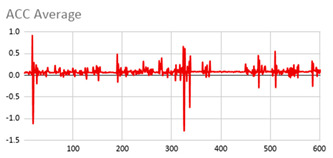	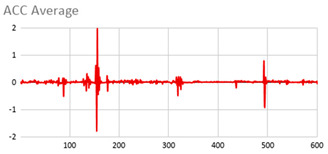
Fuzzy Temporal Window = around 1 s	Fuzzy Temporal Window = around 1 s
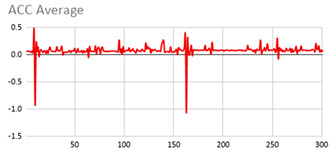	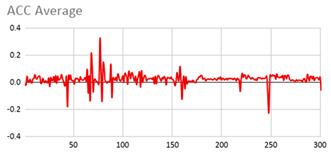
Fuzzy Temporal Window = around 3 s	Fuzzy Temporal Window = around 3 s
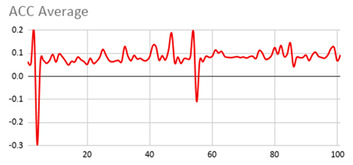	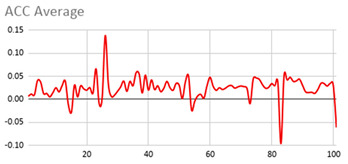
Fuzzy Temporal Window = around 5 s	Fuzzy Temporal Window = around 5 s
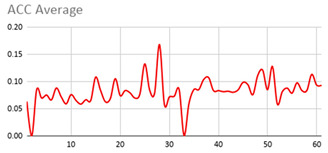	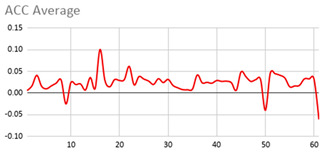

**Table 5:** Rotation (Gyro) results with different fuzzy temporal windows from both wrists.

Rotation (Gyro)	
Dominant Wrist	Non-Dominant Wrist
Fuzzy Temporal Window = around 0.5 s	Fuzzy Temporal Window = around 0.5 s
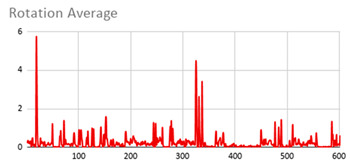	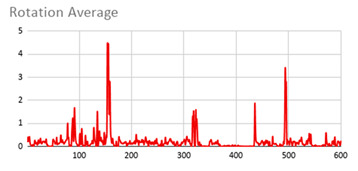
Fuzzy Temporal Window = around 1 s	Fuzzy Temporal Window = around 1 s
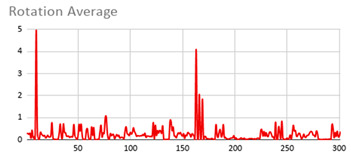	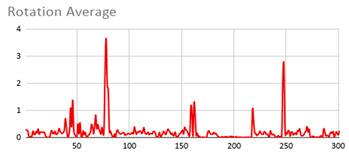
Fuzzy Temporal Window = around 3 s	Fuzzy Temporal Window = around 3 s
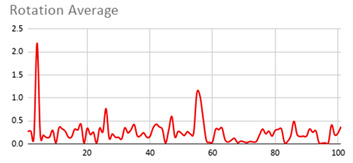	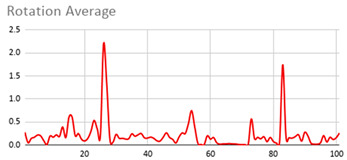
Fuzzy Temporal Window = around 5 s	Fuzzy Temporal Window = around 5 s
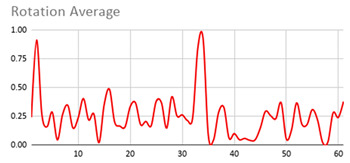	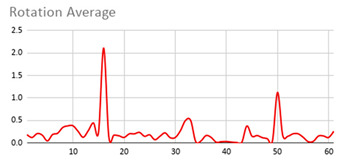

**Table 6:** Heart results with different fuzzy temporal windows from both wrists.

Heart Rate	
Dominant Wrist	Non-Dominant Wrist
Fuzzy Temporal Window = around 1 s	Fuzzy Temporal Window = around 1 s
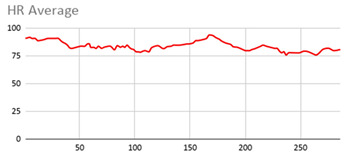	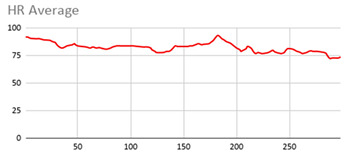
Fuzzy Temporal Window = around 3 s	Fuzzy Temporal Window = around 3 s
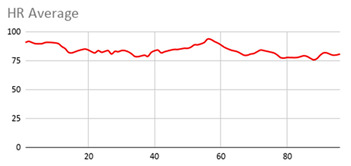	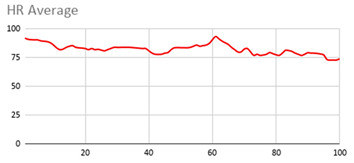
Fuzzy Temporal Window = around 5 s	Fuzzy Temporal Window = around 5 s
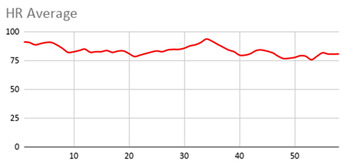	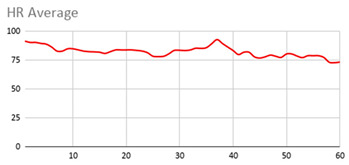

**Table 7:** Sensor data results with six aggregation operators.

Fuzzy Temporal Window = Around 5 s		
Acceleration	Rotation	Heart Rate
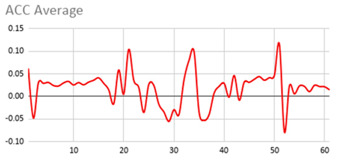	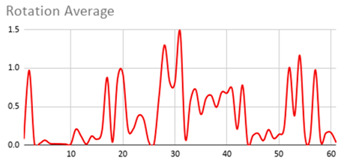	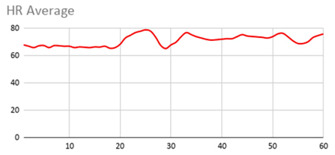
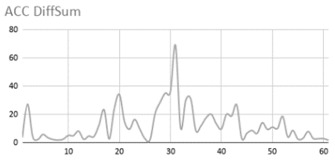	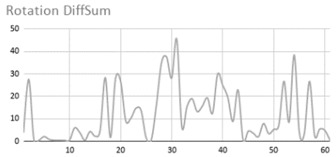	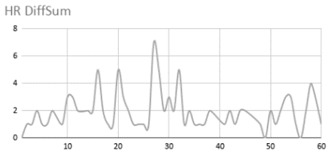
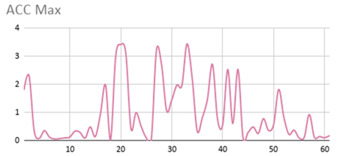	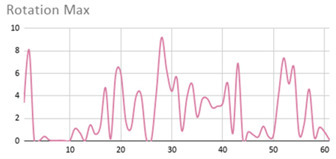	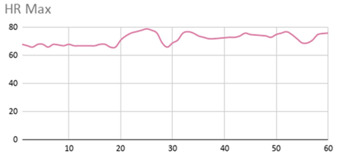
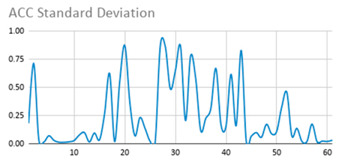	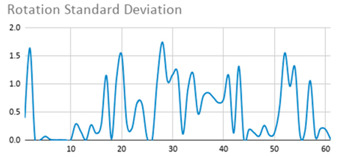	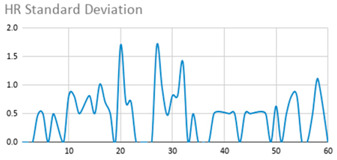
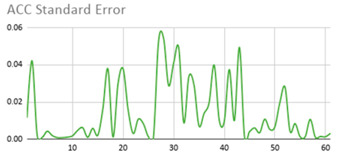	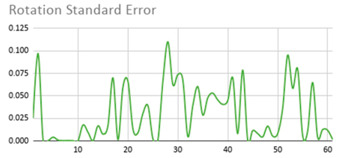	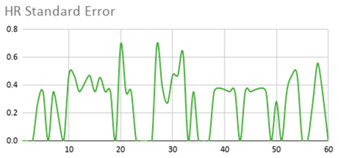
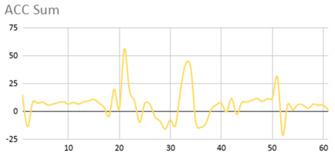	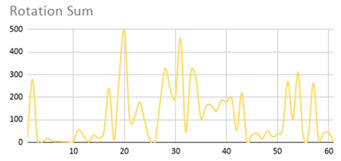	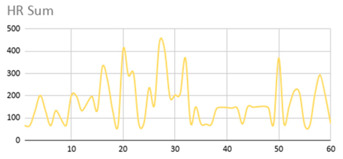

**Table 8:** Evaluation of relevant with three trapezoidal functions.

	Acceleration	Rotation	Heart Rate
Relevant_1	TS(0,0,60,60)	TS(0,0,80,80)	**TS(70,70,100,100)**
Relevant_2	**TS(5,5,70,70)**	TS(0,0,50,50)	TS(60,60,90,90)
Relevant_3	TS(10,10,80,80)	**TS(0,0,65,65)**	TS(60,60,110,110)

The values in bold are the selected by the expert in the evaluation.

**Table 9:** Results of the three relevant linguistic terms for each type of data.

Acceleration	Rotation	Heart Rate
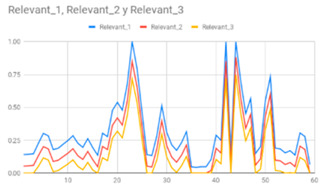	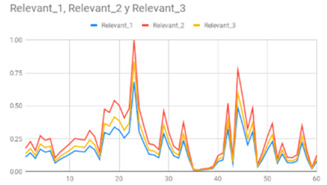	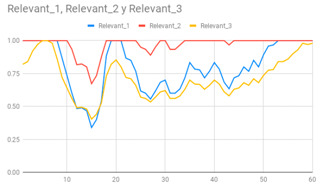

**Table 10:** Membership degree of the PHD of the three people.

	**Person A**	
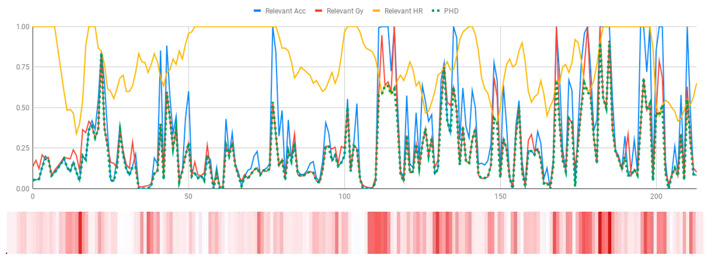		
	**Person C**	
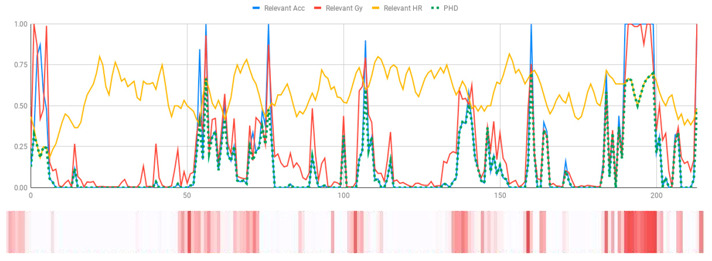		
	**Person D**